# Sclerosing encapsulating peritonitis presenting with paroxysmal abdominal pain and strangulated mechanical bowel obstruction: A case report

**DOI:** 10.1097/MD.0000000000024794

**Published:** 2021-02-26

**Authors:** Hua Tang, Rong Xia, Shuyu Xu, Chenzhe Tao, Chao Wang

**Affiliations:** a Department of General Surgery, Tongling People's Hospital, 468 Bijiashan Road, Tongling, Anhui Province 244000; b Key Lab of Modern Toxicology of Ministry of Education, Center for Global Health, School of Public Health; c State Key Lab of Reproductive Medicine, Institute of Toxicology, Nanjing Medical University, 101 Longmian Avenue, Nanjing, P. R. China.

**Keywords:** Abdominal cocoon, Case report, Etiology, Intestinal obstruction, Sclerosing encapsulating peritonitis

## Abstract

**Rationale:**

Sclerosing encapsulated peritonitis (SEP) is a rare chronic peritoneal inflammation with unknown etiology, and is also known as abdominal cocoon. This occurs when the intestinal annulus is enveloped in the peritoneal cavity, resulting in intestinal obstruction. Its preoperative diagnosis and treatment strategy remains a challenge.

**Patient concerns:**

The study reports a 53-year-old male, who presented with a 4-day history of paroxysmal abdominal pain, without the adverse reaction of nausea, vomiting, or diarrhea.

**Diagnosis:**

The accurate diagnosis of SEP was made after the emergency diagnostic laparoscopy.

**Interventions:**

The laparoscopic exploration revealed that the small intestine was wrapped by a layer of peritoneum. Then, the abdominal fibrous membrane was removed surgically, and adhesiolysis were performed. The patient recovered well, and gradually recovered by the 10^th^ post-operative day.

**Outcomes:**

The patient was discharged uneventfully after 10 days, and the patient recovered well. After the 12-month follow-up, no symptoms of recurrence or complications were observed.

**Lessons:**

The preoperative diagnosis of SEP remains difficult, and the onset of SEP has exhibited a younger trend. The diagnosis of SEP should remain on the list of differential diagnosis for paroxysmal abdominal pain. single-photon emission computed tomography/computed tomography and laparoscopic exploration have been proven to be helpful for establishing the diagnosis. In the early stage of intestinal obstruction caused by SEP, surgical intervention was immediately carried out in emergency department, and the patient recovered well after the operation. The present study also presents a review of the literature for other cases of SEP. The external evidence was helpful in making clinical decisions for patient care.

## Abstract 2

Supplemental Digital Content is available in the text

## 1 Introduction

Sclerosing encapsulating peritonitis (SEP), which is also known as enveloped peritoneal sclerosis (EPS) and encapsulated peritonitis, is a rare clinical syndrome characterized by continuous, intermittent, or repeated bowel obstruction caused by diffuse thickening of peritoneal adhesions.^[2]^ Owtschinnikow was the first to describe the intestinal envelope of fibrous collagen membranes in 1907, and defined this as peritonitis chronic fibrosa incapsulata.^[12]^ Foo *et al.* used the term abdominal cocoon to describe the primary or idiopathic form of this condition, which they noted in adolescent girls from tropical or subtropical countries in 1978.^[3]^ This is more common in young adult females, and has an unclear pathology, while reports on males are quite scanty.^[7,14]^ In addition, due to the lack of specific clinical characteristics, it is not easy to make a timely and effective diagnosis before surgery. SEP usually requires surgical exploration to make a definite diagnosis. Hence, there is a lack of simple and noninvasive means of diagnosis. Nonetheless, recent technological advances in surgical laparoscopic techniques and computed tomography (CT) have made the preoperative diagnosis of SEP possible.^[11]^ The best treatment approach for SEP is complete surgical resection and adhesiolysis. At present, the knowledge on SEP is not very thorough, and more research data needs to be accumulated to deepen the understanding. In present study, one case of SEP treated by the investigators was reported. The present study focused on the aetiopathogenesis, diagnosis and treatment of SEP.

## 2 Case presentation

**Figure 1:**
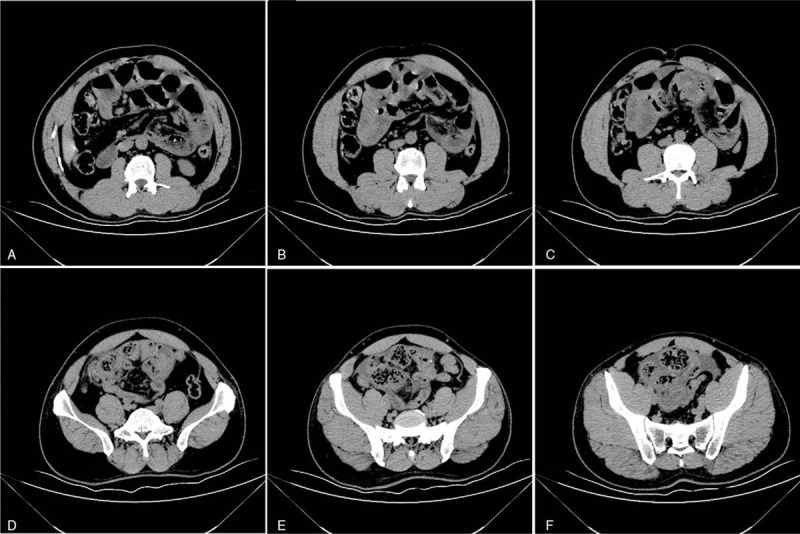
Longitudinal computed tomography images showed typical abdominal cocoon entrapment in the small intestine.

**Figure 2:**
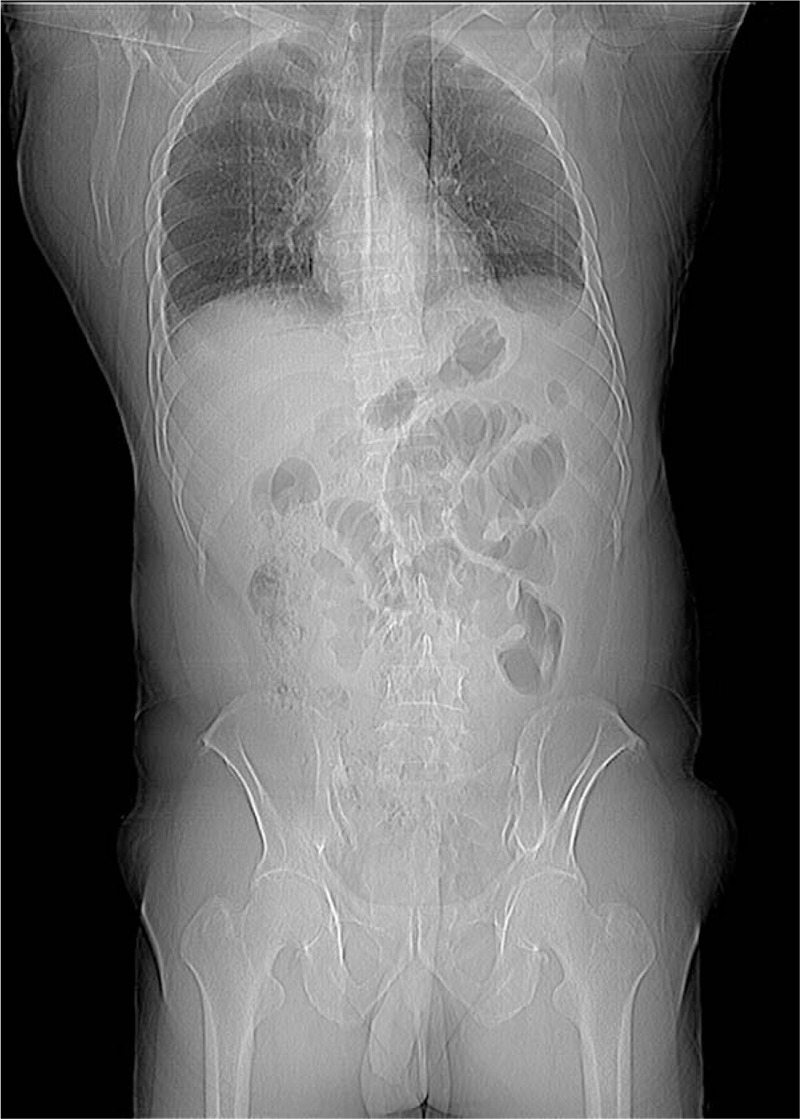
Multiplanar coronal reconstruction computed tomography images showed cauliflower-like appearance of the abdomen and small intestine.

**Figure 3:**
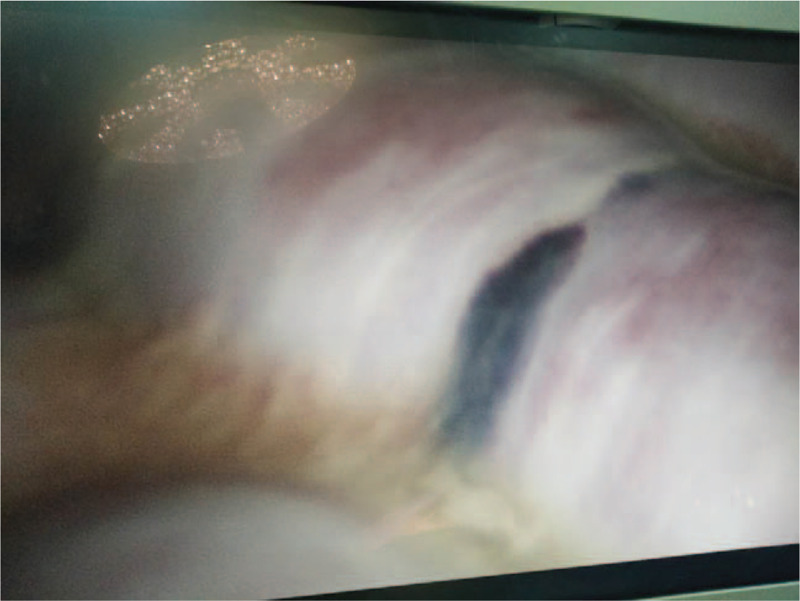
Laparoscopic exploration showed that the small intestine is covered by a membrane-like structure.

**Figure 4:**
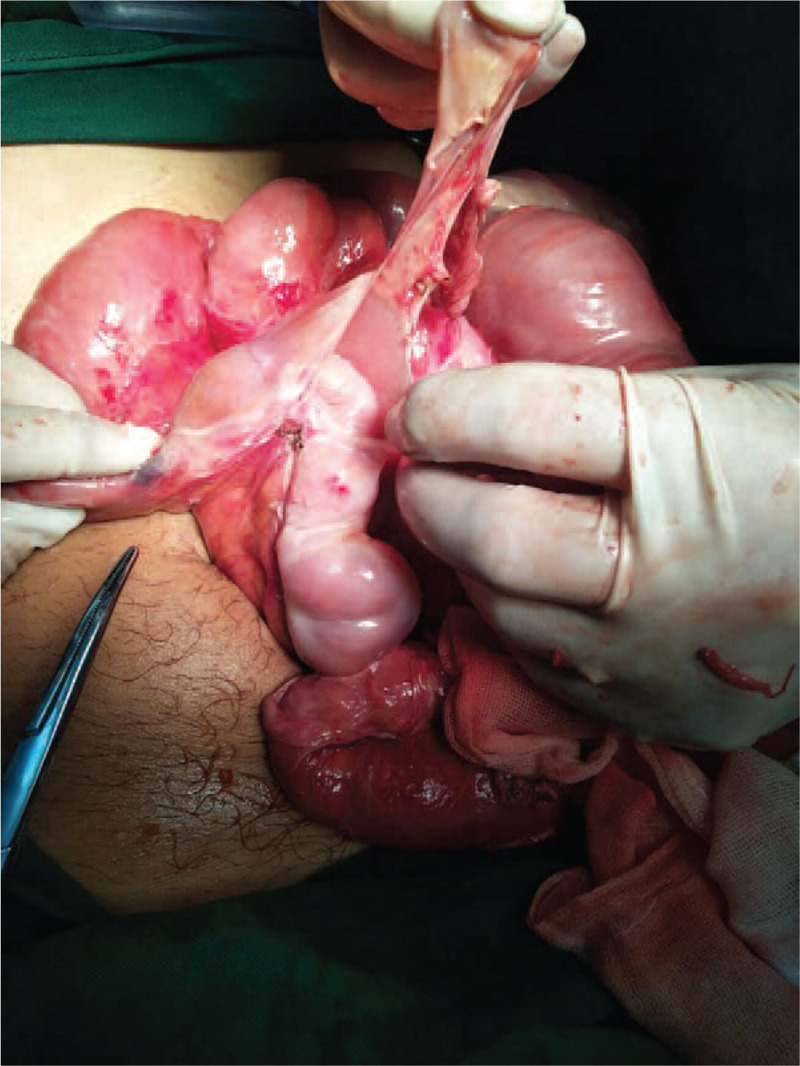
Intraoperative photograph displayed a cocoon-like fashion.

A 53-year-old male patient was admitted to the Emergency Department, and presented with paroxysmal abdominal pain. The abdominal pain occurred without apparent cause for 4 days before admission. The patient had no previous abdominal pain or a history of abdominal surgery. Furthermore, the patient had no significant medical history, such as hypertension, diabetes, or other chronic diseases. The patient was treated by infusion in another hospital for 3 days, but the symptoms of abdominal pain were not relieved, and gradually worsened. The physical examination revealed acute performance, a rapid heart rate of 110 beats/min, abdominal distention, entire abdominal tenderness, and abnormal bowel sounds. The blood routine examination parameters had high peripheral leukocyte counts (19.5 × 10^9^/L) and a high percentage of neutrophils (88%). The abdominal computed tomography detected that the dilated proximal small intestine containing air-fluid (Fig. 1A–C), the intestinal wall of the small intestine was thickened and ischemic, the mesentery density was slightly blurred, and multiple lymph nodes were present. Most of the distal small intestinal tissues were encapsulated by the membrane, and the cross section of the CT imaging was hemispherical (Fig. 1D-F). The preoperative diagnosis of strangulated mechanical intestinal obstruction was diagnosed based on the clinical signs, symptoms, and auxiliary examination (Fig. 2). In emergency surgery, the laparoscopic exploration also suggested the above phenomenon. Furthermore, a little dark red turbid liquid was found in the abdominal cavity, the greater omentum was absent, and the dilated proximal small intestine and distal small intestine covered with membrane-like tissues without spaces were intraoperatively identified (Fig. 3). Due to the difficultly of treatment during the laparoscopic operation, laparotomy was performed. During the operation, the membrane that surrounded the small intestinal was separated, the fiber adhesion between the small intestinal was released, and the necrotic wall of the small intestine intestinal was resected at approximately 20 cm from the ileocecal region. The H&E staining results indicated that this was a fibrous tissue (Fig. 4). For the postoperative anti-inflammatory rehydration support therapy, the patient recovered well without complications, and was discharged from the hospital at 10 days after the operation. During the 12-month follow-up conducted by telephone, the patient's diet and stool were normal, there were no postoperative complications, and the patient lived a normal life. Furthermore, during the follow-up period, the patient had no complaints on the future hospital imaging examinations.

## 3 Literature review

**Figure 5:**
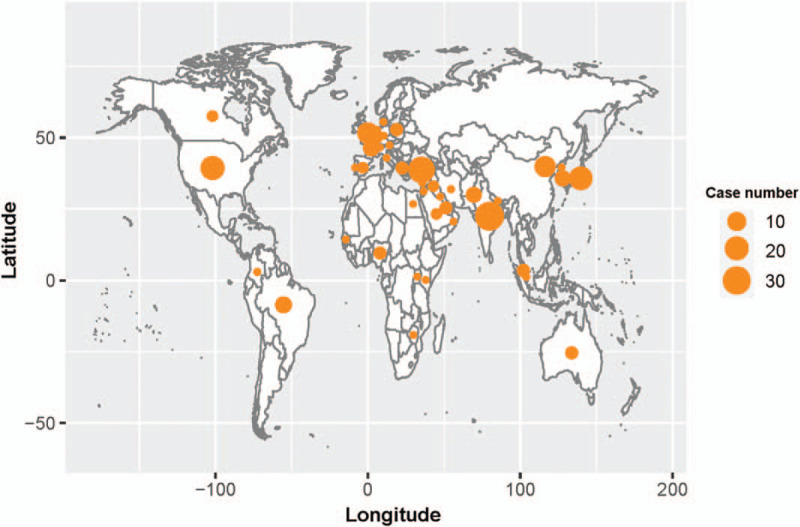
Global distribution of morbidity from 1980 to 2020.

Articles on SEP in the PubMed and Web of Science databases were retrieved from 1980 to 2020, including case reports and reviews, with a total of 206 cases. The investigators used the following key words to identify articles related to SEP: “sclerosing encapsulating peritonitis,” “abdominal cocoon,” and “abdominal cocoon syndrome.” At present, SEP has been consistently reported across populations worldwide, including 43 countries/regions. More than 10 cases have been reported in each country, such as India (33 cases), Turkey (26 cases), USA (20 cases), Japan (19 cases), UK (14 cases), and China (14 cases) (Fig. 5, Data file S1). The male-to-female ratio at presentation was 1.48:1.00. Among these 206 cases, the majority were adult cases (80.6%). Furthermore, more than half of these cases (66.5%) manifested symptoms of intestinal obstruction, and 70 cases (34.0%) were combined with an abdominal mass. The diagnosis of SEP was established based on the intraoperative findings in most cases. In these cases, it was also found that part of the small bowel or the entire small bowel was encapsulated in a gray-white fibrous membrane. In addition, ileal adhesions could be found in some cases. In the present study, 65 cases (31.6%) had complications, which mainly manifested as intestinal fistula (3.9%), peritonitis (4.9%), and ascites (2.4%). SEP can also occur as a complication of other diseases, including diabetes, renal disease, hypertension and chronic liver disease. Almost half of the cases (50.0%) in the present study had an underlying disease, and this was mainly correlated to the liver (14.9%) and kidneys (30.7%). It is noteworthy that 10 cases (4.9%) had SEP secondary to the continuous ambulatory peritoneal dialysis used among the cases with underlying diseases.

## 4 Discussion

SEP is classified as primary (idiopathic) and secondary, according to the underlying etiology. The idiopathic form is also referred to as abdominal cocoon. The causes of idiopathic SEP remains unknown. Most cases of SEP were initially observed in adolescent girls, leading to the hypothesis that retrograde menstruation or retrograde viral infection through the fallopian tubes are potential causes of sclerosing peritonitis.^[1]^ Secondary SEP is more common than idiopathic SEP, which is correlated to many factors. Secondary SEP can be a complication of various clinical treatments, such as abdominal tuberculosis, abdominal surgery, organ transplantation, etc. At present, continuous ambulatory peritoneal dialysis has been widely reported, and SEP can appear as a serious complication of continuous ambulatory peritoneal dialysis.^[18]^ The present literature review also confirms this point. The retrospective analysis suggested that cytoreductive surgery and hyperthermic intraperitoneal chemotherapy are the potential causes of secondary SEP.^[8]^ In addition, SEP is also associated with many diseases, and multiple studies have shown that tuberculosis is an important pathogenic factor for SEP.^[10,13]^ SEP can also occur as rare complications of several diseases, such as hepatitis virus C infection, peritonitis and ascites, severe malnutrition, and so on. Studies have shown that idiopathic SEP occurs most frequently in tropical and subtropical countries, such as China, Malaysia, Singapore, India, Nigeria, Kenya, and South Africa, and idiopathic SEP is twice as common in men, when compared to women.

The manifestations and symptoms of SEP are often vague and non-localized. Patients often present with vomiting, abdominal pain, or abdominal distension and other symptoms of subacute intestinal obstruction.^[21]^ The condition becomes progressively worse if not treated in time, and the consequences would become more serious. Laboratory tests for SEP are nonspecific, and are associated with underlying infections, malnutrition, and inflammation. At present, no specific and effective biomarkers have been found. Due to lack of specific clinical manifestations and testing indexes, an accurate and prompt preoperative diagnosis remains difficult. In recent years, advanced imaging techniques, especially multi-slice CT scans, have helped to achieve the pre-operative diagnosis. The CT revealed that the small intestinal loops congregate to the center of the abdomen covered with fibrous membrane.^[19]^ Other imaging features include calcification, localized effusion, and lumpy small intestine.^[20]^ The histological examination of the thick membrane is always fibrous tissue, with or without chronic inflammatory cell infiltration. Laparoscopy, ultrasound, and magnetic resonance imaging (MRI) can also provide clues to the diagnosis.^[15]^ MRI can reveal the local signal enhancement, and positron emission tomography can reveal the diffuse peritoneal implants. Meanwhile, diagnostic laparoscopy can reveal the peritoneal and omental implants.^[5,16]^ Therefore, the typical CT or MRI findings, along with the histopathology finding of fibrocollagenous tissue with an inflammatory infiltrate, provide the complete diagnosis of SEP.

SEP treatment should be based on the patient's condition, when choosing the appropriate treatment. Patients with mild symptoms may receive conservative treatment, such as nutritional support, gastrointestinal decompression, and medication. The renin-angiotensin-aldosterone system inhibitor therapy and anti-inflammatory agents, including colchicine and immunosuppressants (such as mycophenolate mofetil, azathioprine, cyclosporine, etc), have been found to be efficacious for the treatment of SEP.^[17]^ In addition, corticosteroids and tamoxifen can also alleviate symptoms and restore bowel function in patients with SEP.^[2]^ Hirahara *et al.* successfully established a rat model of sclerosing peritonitis induced by the intraperitoneal injection of chlorhexidine gluconate and methylglyoxal, which can be used to analyze the mechanism of progression of peritoneal injury and EPS.^[4]^ Ju *et al.* applied this model, and reported that a novel AMP-activated protein kinase activator (HL156A) can reduce the degree of peritoneal fibrosis caused by chlorhexidine gluconate, suggesting that HL156A has clinical value in the treatment of SEP.^[6]^ Surgery remains as the gold standard treatment for SEP. Surgical treatment is performed mainly through the release of the fibrous capsule and small intestinal adhesion, thereby relieving the intestinal obstruction. A retrospective analysis of 12 cases revealed that compared with simple membrane excision, partial resection, enterectomy and exploratory laparotomy, membrane excision can reduce the patient's persistent intestinal obstruction or mortality.^[9]^ Surgical therapy is not recommended for patients with ascites, asymptomatic sclerosing peritonitis, or subacute intestinal obstruction.^[20]^

In conclusion, the preoperative diagnosis of SEP remains very difficult due to the lack of specificity in the early clinical manifestation. The present study has listed many potential causes of SEP, and described the main symptoms and diagnostic basis, aiming to raise the people's awareness of the disease. single-photon emission computed tomography/CT and laparoscopic exploration have been proven to be helpful for establishing the diagnosis. At present, the understanding of the pathogenesis of SEP remains largely inadequate. Therefore, effective interventions cannot be implemented at the early stage of the disease, except for surgical resection. The key to avoid surgical injury is to identify the etiology, early diagnosis and early treatment, which needs further study.

## Acknowledgment

The authors are very grateful to the patient for his consent.

## Author contributions

**Conceptualization:** Hua Tang, Rong Xia, Shuyu Xu, Chenzhe Tao, Chao Wang.

**Data curation:** Hua Tang, Rong Xia, Shuyu Xu.

**Formal analysis:** Rong Xia, Shuyu Xu, Chenzhe Tao.

**Funding acquisition:** Hua Tang, Chao Wang.

**Investigation:** Hua Tang, Rong Xia, Shuyu Xu.

## Supplementary Material

Supplemental Digital Content
